# Development and validation of a prognostic model for neurological deterioration in acute posterior circulation cerebral infarction

**DOI:** 10.1186/s12883-026-04823-7

**Published:** 2026-03-17

**Authors:** Zedan Guo, Qian Zhang, Jiani Wu, Li Yi

**Affiliations:** 1 PKU-Shenzhen Clinical Institute of Shantou University Medical College, Shenzhen, Guangdong province China; 2 Department of Neurology, Peking University Shenzhen Hospital, Peking University, Lianhua Road 1120, Futian District, Shenzhen, Guangdong Province CN-518036 China; 3 School of Public Health, Shenzhen University Medical School, Shenzhen, Guangdong province China; 4 Department of Medical Administration, Peking University Shenzhen Hospital, Peking University, Shenzhen, Guangdong province China

**Keywords:** Prognosis model, Nomogram, Cerebral Infarction, Posterior Circulation, Neurological Deterioration

## Abstract

**Background:**

Neurological deterioration occurs in 8%-40% of patients with acute posterior circulation cerebral infarction (APCCI), leading to higher disability and mortality. We developed and validated a prognostic model integrating dynamic physiological indicators and quantitative neuroimaging indicators for early risk stratification.

**Methods:**

We retrospectively enrolled patients with APCCI from Peking University Shenzhen Hospital into training set (*n* = 447) and testing set (*n* = 94). The model incorporates quantitative neuroimaging (mean apparent diffusion coefficient value (MAV) of infarct lesions) and dynamic physiological monitoring (systolic blood pressure variability), combining qualitative and quantitative approaches to better characterize infarction and hemodynamic instability. A nomogram was constructed and evaluated using discrimination, calibration, and clinical decision curve analysis (DCA).

**Results:**

The model demonstrated moderate discrimination, with an AUC of 0.74 (95% CI: 0.62–0.86) in the testing set, and ideal calibration (Brier score 0.15). DCA confirmed superior clinical benefit within an 8%–56% threshold range. Both MAV and blood pressure variability were significant predictors, highlighting their added prognostic value beyond conventional factors.

**Conclusions:**

The prognostic model effectively identifies APCCI patients at risk of neurological deterioration by incorporating innovative neuroimaging and dynamic monitoring indicators. Temporal validation shows promising performance in the validation cohort, and further multi-center validation is warranted for clinical adoption.

**Supplementary Information:**

The online version contains supplementary material available at 10.1186/s12883-026-04823-7.

## Introduction

Acute posterior circulation cerebral infarction (APCCI) accounts for approximately 20% of all cerebral infarction cases. Notably, 8%-40% of APCCI patients experience neurological deterioration (ND) despite early administration of evidence-based therapies such as antiplatelet agents and statins [1, 2]. Compared to anterior circulation infarction, APCCI exhibits higher disability, mortality rates and more insidious clinical presentation, which often delays timely intervention and severely impacts patient outcomes.

Current studies mainly focus on exploring the investigated predictors of ND in acute ischemic stroke (AIS). Research has found the neutrophil-to-lymphocyte ratio (NLR) as a crucial biomarker that reflects the dynamic balance between early cerebral injury and neuroprotective mechanisms [3]. Emerging evidence also indicates that reduced mean apparent diffusion coefficient values (MAV) in ischemic regions demonstrate significant correlations with infarction irreversibility and elevated risks of secondary brain injury [4, 5]. Furthermore, baseline National Institutes of Health Stroke Scale (NIHSS) scores, a validated measure of stroke severity, effectively predicted ND risk [6]. Additional meaningful predictors include age, gender, dyslipidemia (particularly low density lipoprotein cholesterol [LDL-C] and triglyceride [TG]) [7–9]. Nevertheless, conventional single-parameter prediction systems show suboptimal discriminative capacity [4, 9]. Current predictive models primarily focus on anterior circulation or pontine infarction cohorts and mostly lack robust external validation. These limitations highlight the critical need to develop and validate integrated multivariable prediction models for precise ND risk stratification in APCCI populations.

Prognostic models, constructed via logistic regression or advanced methods (e.g., machine learning), quantify variable-specific predictive contributions and are often implemented through nomogram or digital tools to enable early intervention [10, 11].

## Methods

### Study design and participants

We enrolled patients diagnosed with APCCI who were hospitalized at Peking University Shenzhen Hospital between 1 January 2019 and 1 March 2024 as the training cohort, which was used for model construction and internal validation. Meanwhile, a validation cohort comprising patients with confirmed APCCI from the same center but in a different time period (i.e., APCCI diagnosed between 1 March 2023 and 1 March 2025) was recruited for subsequent temporal validation of the model [11]. All enrolled patients met the following criteria. Inclusion criteria: (a) Patients admitted ≤ 24 h post-symptom onset received standardized antiplatelet and statin therapy; (b) Age ≥ 18 years; (c) No gender-based restriction.Exclusion criteria: (a) Clinical data missing by more than 30%; (b) Receiving thrombolysis or endovascular interventions; (c) Anterior circulation infarction, hemorrhagic stroke, or transient ischemic attack (TIA); (d) Inability and refusal to assess neurological deficits within 7 days of admission; (e) Concomitant severe infections, malignancies, severe cardiopulmonary diseases, hepatic or renal failure, or hematologic disorders.

### Data collection and processing

We collected the following data within the first 24 h of hospitalization: demographics (age, gender, stroke risk factors [SRF; including hypertension, diabetes, dyslipidemia, smoking, hyperhomocysteinemia, prior stroke, atrial fibrillation, valvular heart disease and obesity]), clinical parameters (baseline NIHSS scores [BNS], onset time [OT], systolic and diastolic blood pressure variability [SBPV and DBPV] and infarction subtype [IS]), along with laboratory and imaging data. We divided stroke risk factors (SRF) into four categories according to the number: none (0), mild (1–2), moderate (3–4), severe (≥ 5). OT was stratified into 6 parts by 4-hour increments. We defined hypertension as systolic blood pressure ≥ 140 mmHg, diastolic blood pressure ≥ 90 mmHg or active antihypertensive treatment; Diabetes was defined as fasting plasma glucose ≥ 7 mmol/L twice or active hypoglycemic agents use; Dyslipidemia criteria: Total cholesterol (TC) ≥ 5.7 mmol/L or TG ≥ 1.7 mmol/L twice or hypolipidemic treatment; Smokers were defined as individuals consuming ≥ 1 cigarette daily for ≥ 6 months; Hyperhomocysteinemia was defined as plasma homocysteine ≥ 18 µmol/L; For the calculation of blood pressure variability, blood pressure was measured once on the day of admission and at least four times daily thereafter, with relatively consistent measurement times. Therefore, we collected multiple systolic and diastolic blood pressure readings for each patient after admission, calculated their mean and standard deviation respectively, and finally defined systolic blood pressure variability (SBPV) and diastolic blood pressure variability (DBPV) as the ratio of standard deviation to mean.Since the time of neurological deterioration varied among patients, to ensure that variable inclusion was not affected by the outcome of neurological deterioration, we censored the blood pressure data. Specifically, only blood pressure measurements obtained before the occurrence of neurological deterioration were used to calculate SBPV and DBPV [12]. We classified infarction subtype (IS) into 5 groups by conforming to the Trial of Org 10,172 in Acute Stroke Treatment (TOAST) criteria and then merged “other determined etiology” with “undetermined etiology” categories due to low case frequencies [13].

All participants underwent magnetic resonance imaging (MRI) using a 3.0T Siemens Sigma system (Siemens Healthineers). Diffusion-weighted imaging (DWI) acquisitions used single-shot EPI sequences (TE/TR = 88/2700 ms, flip angle = 90°, matrix = 128 × 128, field of view = 250 × 250 mm², resolution = 1 × 1 mm²). We applied three b-values (maximum 1000 s/mm²) to capture DWI images under three diffusion sensitivities. Section parameters included 4 mm slice thickness and 4.8 mm gap. The system automatically generated apparent diffusion coefficient (ADC) maps after we selected ADC imaging parameters. For ADC quantification, we imported DICOM-format DWI and ADC images into 3D-Slicer software (v5.6.2). We manually delineated infarct regions on ADC maps. The software then identified ischemic lesions and automatically calculated both the MAV within the delineated voxels and the total infarct volume [14]. All imaging data were acquired prior to neurological deterioration to avoid confounding effects. We addressed missing data through multiple imputation following TRIPOD and PROBAST guidelines, with complete imputed datasets provided in Supplementary files [15, 16].

### Definition of neurological deterioration

Neurological deterioration (ND) was diagnosed when NIHSS total score increased by ≥ 2 points or motor subscores increased by ≥ 1 point during the first 7 hospitalization days, despite standardized antiplatelet and statin therapy [17]. Trained and certified neurologists performed NIHSS assessments on admission, with daily reassessments conducted for 7 consecutive days. All clinical staff remained masked to study allocation throughout the entire study.

### Sample size calculation

Sample size estimation using the pmsampsize package (R studio v4.5.0) accounted for 14 candidate predictors, an expected C-statistic of 0.85 and a 25% expected event rate. The minimum requirement of 373 participants was exceeded by enrolling 447 cases. The training and validation sets were split at approximately 5:1 [10, 18].

### Statistic analysis

**Fig. 1:**
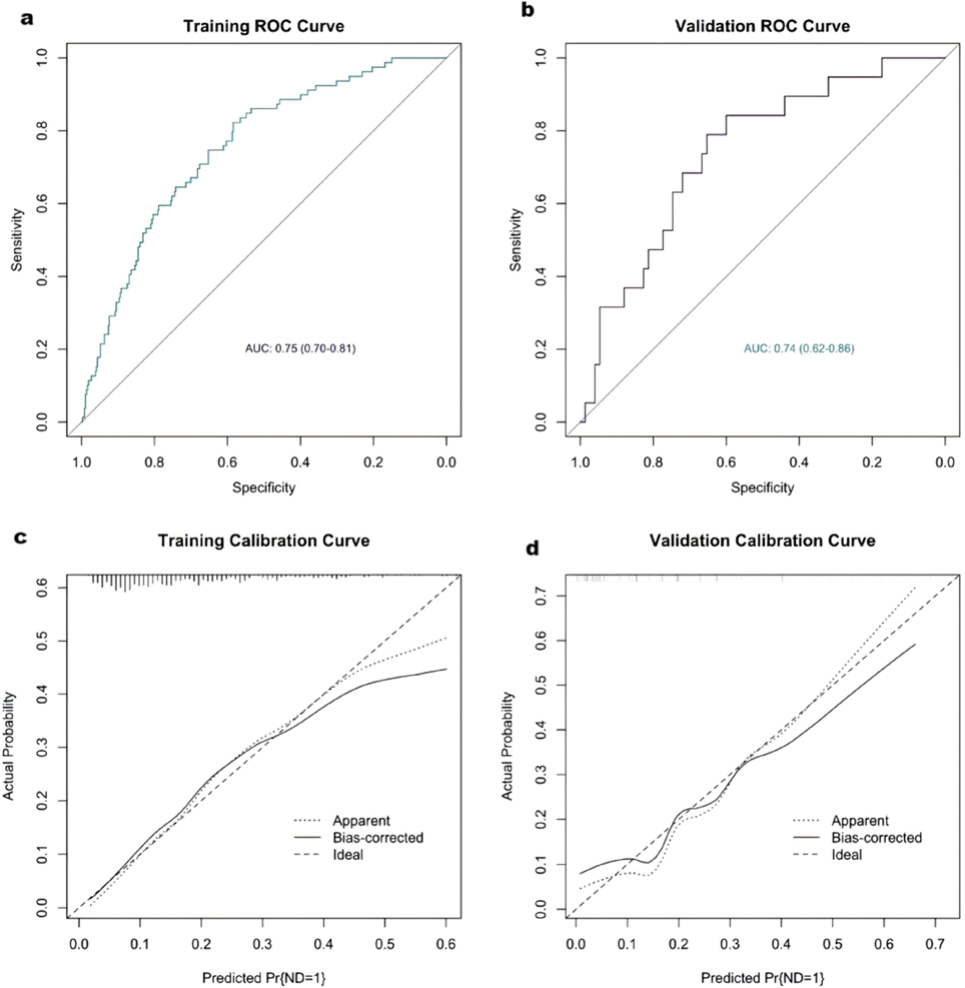
Receiver operating characteristic (ROC) and calibration curves. Legend text: (**a**) ROC curves of the training set and (**b**) the testing set; (**c**) Calibration curve of the training set (Brier score = 0.13) and (**d**) the temporal validation set (Brier score = 0.15)

Statistical analyses utilized R studio (v4.5.0). Continuous variables are shown as mean ± SD or median (IQR), categorical variables are shown as percentage (%). Inter-group comparisons employed parametric or nonparametric tests for continuous data and chi-square or Fisher’s exact tests for categorical data. For variables with less than 30% missing data, we performed multiple imputation and normalized their distributions, whereas variables with more than 30% missing data were excluded (shown in supplementary Tables 1 and Table 2). In order to minimize overfitting and select robust predictors to develop the model, we performed least absolute shrinkage and selection operator (LASSO) regression. Prior to performing LASSO regression, we conducted a correlation analysis on all variables and constructed a correlation heatmap (see Fig. 1). We observed no strong correlations between the variables, except for a moderate correlation between SBPV and DBPV. Model discrimination was quantified by the AUC, with values approaching 1.0 signifying superior predictive accuracy. We assessed model calibration using calibration curves and Brier scores. Closer alignment of the curve to the 45° line and lower Brier scores demonstrated better calibration performance. We internally validated the model’s reproducibility using 1,000 bootstrap resamples [19]. Temporal validation in the cohort tested the model’s generalizability. We quantified clinical utility via DCA, calculating net benefits across threshold probabilities. This study adhered to the TRIPOD guidelines (Transparent Reporting of a Multivariable Prediction Model for Individual Prognosis Or Diagnosis).

## Results

### Participants

Following the inclusion and exclusion criteria, 447 patients (mean age 63.90 ± 13.83 years; 70.0% male) and 94 patients (mean age 62.11 ± 11.59 years; 73.4% male) with APCCI were enrolled in the training and testing sets, respectively. Baseline characteristics of both cohorts are summarized in Table 1. The two sets demonstrated no significant difference in ND incidence (17.7% vs. 20.2%, *P =* 0.664). Statistically significant differences (*P* < 0.05) were only observed in infarct volume (IV) (*P* = 0.027).

**Table 1:** Baseline characteristics in the training and testing sets

Variables	Neurological Deterioration (*n* = 98)	Non-Neurological Deterioration (*n* = 443)	*P*-value
Gender			0.596
* Female*	134 (30.0%)	25 (26.6%)	
* Male*	313 (70.0%)	69 (73.4%)	
Age	63.90 ± 13.83	62.11 ± 11.59	0.190
SBPV	0.08 ± 0.03	0.08 ± 0.03	0.612
DBPV	0.09 ± 0.04	0.09 ± 0.03	0.577
Baseline NIHSS scores	2.90 ± 2.97	2.77 ± 3.28	0.723
Neurological Deterioration	79 (17.7%)	19 (20.2%)	0.664
Onset time			0.165
* 0:00–04:00*	51 (12.0%)	9 (9.7%)	
* 04:00–08:00*	65 (15.3%)	22 (23.7%)	
* 08:00–12:00*	84 (19.7%)	23 (24.7%)	
* 12:00–16:00*	59 (13.8%)	14 (15.1%)	
* 16:00–20:00*	86 (20.2%)	14 (15.1%)	
* 20:00–24:00*	81 (19.0%)	11 (11.8%)	
NLR	3.99 ± 4.05	4.70 ± 4.59	0.171
LDL-C	3.19 ± 0.89	3.39 ± 1.10	0.091
Infarct volume	2,071.38 ± 6,642.88	5,090.78 ± 11,473.99	0.027
MAV	474.35 ± 76.38	483.31 ± 81.84	0.375
Pontine infarction			>0.999
* No*	260 (59.2%)	54 (58.7%)	
* Yes*	179 (40.8%)	38 (41.3%)	
Infarction subtype			0.084
* Large-artery atherosclerosis*	117 (30.1%)	37 (44.0%)	
* Cardioembolism*	32 (8.2%)	2 (2.4%)	
* Small-vessel occlusion*	216 (55.5%)	41 (48.8%)	
* Other determined etiology*	6 (1.5%)	1 (1.2%)	
* Undetermined etiology*	18 (4.6%)	3 (3.6%)	
Stroke risk factors			0.637
* 0 (none)*	43 (9.6%)	8 (8.5%)	
* 1 (mild)*	235 (52.6%)	45 (47.9%	
* 2 (moderate)*	150 (33.6%)	38 (40.4%)	
* 3 (severe)*	19 (4.3%)	3 (3.2%)	
TG	1.63 ± 1.13	1.60 ± 0.82	0.773

*SBPV *systolic blood pressure variability, *DBPV *diastolic blood pressure variability, *NIHSS *National Institutes of Health Stroke Scale, *NLR *neutrophil-to-lymphocyte ratio, *MAV *mean apparent diffusion coefficient value, *LDL-C *low density lipoprotein cholesterol, *TG *triglyceride

Table 2 compares baseline characteristics between ND and non-ND (NND) patients. Variables potentially associated with ND (*P* < 0.1 on univariate analysis) included age, baseline NIHSS scores (BNS), NLR, MAV, IS and PI.

**Table 2:** Comparison of two groups: Neurological Deterioration and Non-Neurological Deterioration

Variables	Neurological Deterioration (*n* = 98)	Non-Neurological Deterioration (*n* = 443)	*P*-value
Gender			0.941
* Female*	28 (28.6%)	131 (29.6%)	
* Male*	70 (71.4%)	312 (70.4%)	
Age	66.28 ± 12.03	63.00 ± 13.71	0.019
SBPV	0.09 ± 0.03	0.08 ± 0.03	0.113
DBPV	0.09 ± 0.04	0.09 ± 0.04	0.525
Baseline NIHSS scores	4.14 ± 2.94	2.59 ± 2.98	< 0.001
Onset time			0.622
* 0:00–04:00*	11 (11.5%)	49 (11.6%)	
* 04:00–08:00*	19 (19.8%)	68 (16.1%)	
* 08:00–12:00*	22 (22.9%)	85 (20.1%)	
* 12:00–16:00*	16 (16.7%)	57 (13.5%)	
* 16:00–20:00*	14 (14.6%)	86 (20.3%)	
* 20:00–24:00*	14 (14.6%)	78 (18.4%)	
NLR	5.14 ± 5.83	3.89 ± 3.65	0.044
LDL-C	3.25 ± 0.96	3.22 ± 0.93	0.746
Infarct volume	2,514.03 ± 9,902.17	2,602.62 ± 7,160.67	0.937
MAV	447.30 ± 78.90	482.67 ± 75.47	< 0.001
Pontine infarction			< 0.001
* No*	40 (41.2%)	274 (63.1%)	
* Yes*	57 (58.8%)	160 (36.9%)	
Infarction subtype			0.093
* Large-artery atherosclerosis*	32 (36.4%)	122 (31.7%)	
* Cardioembolism*	3 (3.4%)	31 (8.1%)	
* Small-vessel occlusion*	52 (59.1%)	205 (53.2%)	
* Other determined etiology*	1 (1.1%)	6 (1.6%)	
* Undetermined etiology*	0 (0.0%)	21 (5.5%)	
Stroke risk factors			0.324
* 0 (none)*	5 (5.1%)	46 (10.4%)	
* 1 (mild)*	57 (58.2%)	223 (50.3%)	
* 2 (moderate)*	32 (32.7%)	156 (35.2%)	
* 3 (severe)*	4 (4.1%)	18 (4.1%)	
TG	1.64 ± 1.16	1.62 ± 1.06	0.922

*SBPV *systolic blood pressure variability, *DBPV *diastolic blood pressure variability, *NIHSS *National Institutes of Health Stroke Scale, *NLR *neutrophil-to-lymphocyte ratio, *MAV *mean apparent diffusion coefficient value, *LDL-C *low density lipoprotein cholesterol, *TG *triglyceride

## Model development and performance

**Fig. 2:**
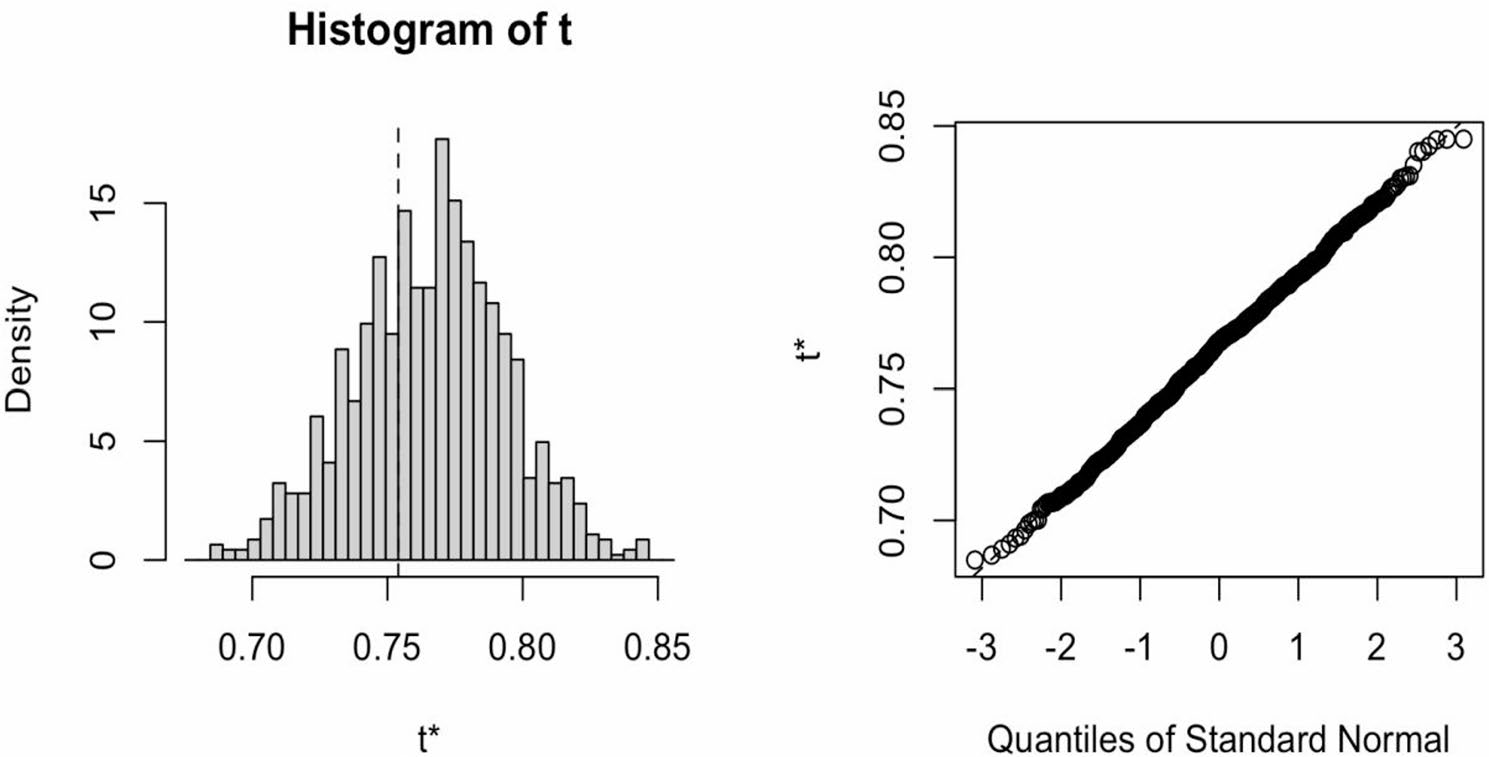
Bootstrap internal validation. Legend text: The model was validated with 1000 bootstrap replicates. The discrimination metric (C-index = 0.754) showed minimal bias (bias = 0.011) and standard error (std.error = 0.028). The calibration metric (Brier score = 0.129) demonstrated near-zero bias (bias = -0.004) and standard error (std.error = 0.011), suggesting low overfitting risk and excellent generalizability

**Fig. 3:**
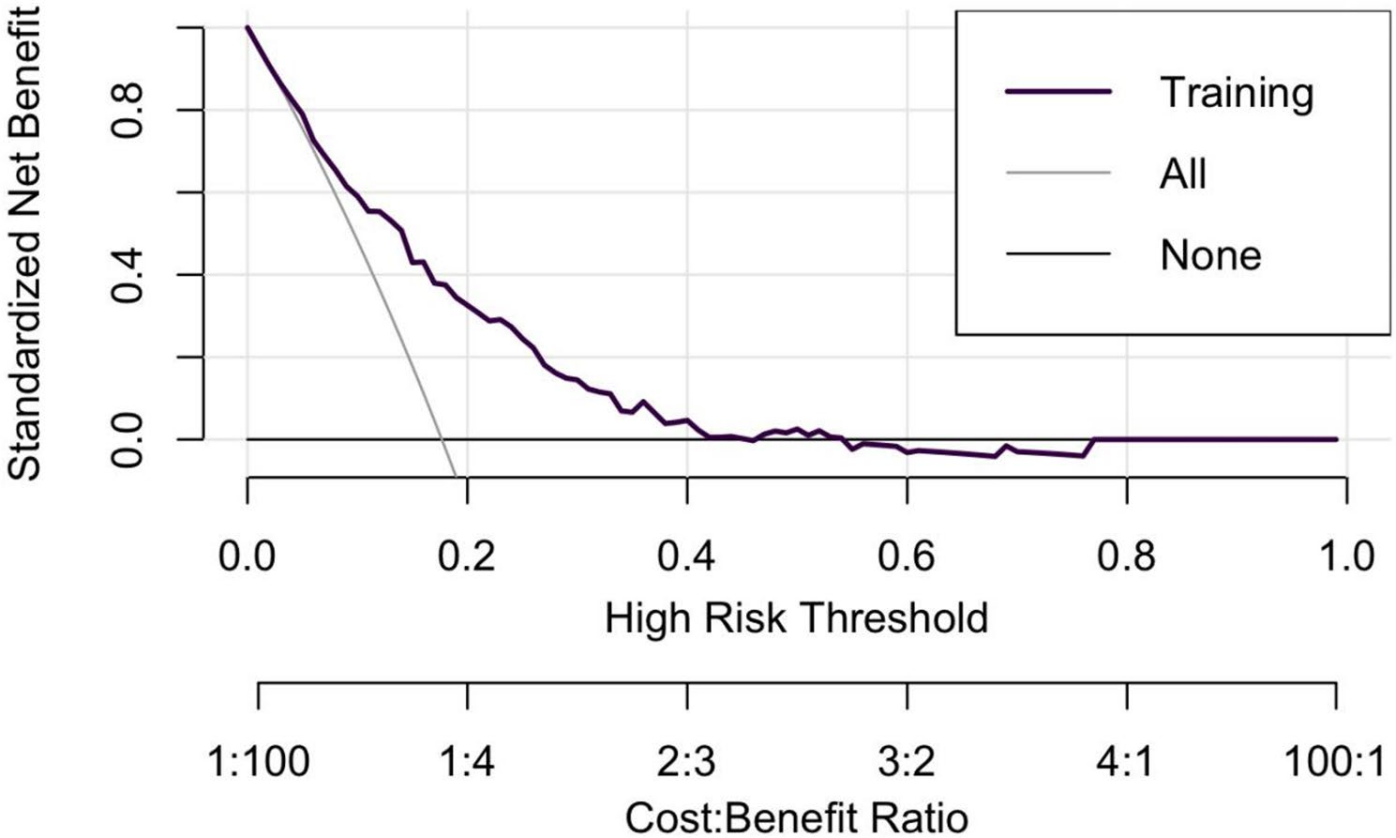
Decision curve analysis. Legend text: The model was validated with 1000 bootstrap replicates. The discrimination metric (C-index = 0.754) showed minimal bias (bias = 0.011) and standard error (std.error = 0.028). The calibration metric (Brier score = 0.129) demonstrated near-zero bias (bias = -0.004) and standard error (std.error = 0.011), suggesting low overfitting risk and excellent generalizability

LASSO regression analysis in the training set identified optimal performance at **λ** = 0.016. The coefficient path plot and cross-validation curve of the LASSO regression are provided in the Supplementary Materials (see Figs. 2 and 3**)**. We also provided the complete formula of the model intercept and all regression coefficients as follow:

$$\begin{aligned}\mathrm{N}\mathrm{e}\mathrm{u}\mathrm{r}\mathrm{o}\mathrm{l}\mathrm{o}\mathrm{g}\mathrm{i}\mathrm{c}\mathrm{a}\mathrm{l}\mathrm{D}\mathrm{e}\mathrm{t}\mathrm{e}\mathrm{r}\mathrm{i}\mathrm{o}\mathrm{r}\mathrm{a}\mathrm{t}\mathrm{i}\mathrm{o}\mathrm{n}&=(-2.374389404)+0.007068815\ast\mathrm{a}\mathrm{g}\mathrm{e}\\&+2.808204171\ast\mathrm{S}\mathrm{B}\mathrm{P}\mathrm{V}+0.799761268\ast\mathrm{B}\mathrm{N}\mathrm{S}\\&-0.039872893\ast\mathrm{O}\mathrm{T}+0.279378682\ast\mathrm{N}\mathrm{L}\mathrm{R}\\&-0.002644469\ast\mathrm{M}\mathrm{A}\mathrm{V}+0.208360197\ast\mathrm{P}\mathrm{I}\end{aligned}$$

**Fig. 4:**
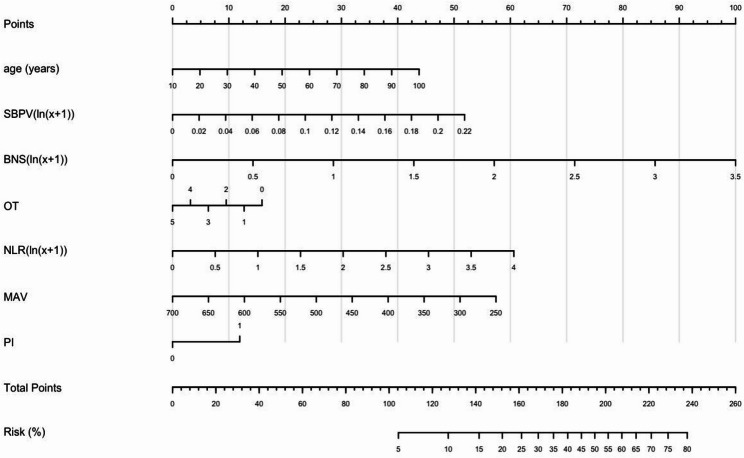
START nomogram for predicting neurological deterioration in patients with acute posterior circulation cerebral infarction

The training set exhibited an AUC of 0.75 (95% CI: 0.70–0.81), suggesting moderate and acceptable discriminative performance (shown in Fig. 1a). Figure 1c displays the calibration curve of the model (Brier score: 0.13). We performed internal validation through 1000 bootstrap resamples within the training set (shown in Fig. 2). The model’s discriminative performance showed minimal bias (bias = 0.012) and standard error (standard error = 0.028). Its calibration performance also demonstrated very low overfitting risk (bias = -0.004; standard error = 0.011). Temporal validation was further conducted using an independent testing set to evaluate the model’s generalizability (AUC = 0.74, 95%CI: 0.62–0.86; Brier score: 0.15) (Fig. 1b and d) [20]. The DCA (Fig. 3) of the model demonstrated significant clinical utility. Within a threshold probability range of 8% to 56%, the model-guided strategy (intensified therapy for high-risk patients) provided greater net benefit than “treat all” or “treat none” approaches. Similarly, we also observed comparable performance in the validation set (see Fig. 4). Finally, we constructed a nomogram **(**Fig. 4**)** to visually demonstrate the impact and contribution of each predictor.

## Discussion

Annually, those patients with APCCI-related ND always suffer significant declines in long-term quality of life and exhibit higher mortality rates compared to non-progressing cases. Currently, predictive models for APCCI-related ND remain scarce, and most lack external validation, limiting their clinical reliability [4, 21]. Our study developed a prognostic model incorporating seven variables: age, BNS, SBPV, NLR, OT, PI and MAV. The model exhibits relatively favorable discriminative and calibrated accuracy (testing set AUC = 0.74, 95%CI: 0.62–0.86; Brier score = 0.15), which exerts a moderate indicative effect for clinicians to early identify high-risk patients with APCCI and implement personalized interventions to reduce the risk of ND.

To contextualize our model’s performance against existing ND predictive tools, we directly compared it with key published models across four dimensions: target cohort, predictor composition, discriminative efficacy, and validation rigor. For posterior circulation stroke subgroup models, one study [22] developed a clinical-only ND prediction model for acute pontine infarction (a core APCCI subset) with an AUC of 0.84, which was higher than our model’s 0.74. However, their model did not include neuroimaging markers and only completed internal validation, whereas our model integrated clinical, inflammatory, and imaging indicators and underwent 1,000-fold bootstrap internal validation (calibration bias=-0.004) and temporal external validation, helping to reduce potential overfitting risks. The other study [4] constructed an imaging-only model for isolated pontine infarction (based on MAV signatures) with an AUC of 0.71, which had slightly lower discriminative performance than our model, as it failed to incorporate clinical and inflammatory predictors (e.g., SBPV, NLR) that reflect the multifactorial pathophysiology of APCCI-related ND.

The incidence of ND in our training and validation cohorts (17.7% and 20.2%, respectively) aligns with prior reports in APCCI populations. Consistent with established evidence, age, and BNS were confirmed as predictors of ND [6–9, 23]. Our model further highlights SBPV as a critical contributor, where both hypotension (reducing penumbral perfusion) and hypertension (exacerbating edema and reperfusion injury) disrupt cerebral autoregulation [12]. The NLR is a significant marker of systemic inflammation. Neutrophil infiltration amplifies local oxidative stress and thrombogenesis through cytokine release and endothelial damage, while lymphopenia reflects stress-induced immunosuppression, impairing tissue repair. These processes likely synergize to accelerate secondary brain injury, consistent with our findings. Our study found that onset time (OT) exhibited a negative coefficient during model construction. At first glance, this result may seem counterintuitive, but in fact, from the perspectives of our study’s coding system and modeling methodology, this result is consistent with the circadian rhythm characteristics of stroke and statistical principles reported in existing published literature.In our study, OT refers to the circadian time window of each patient’s stroke onset, and we adopted ordinal coding for it: the whole day was divided into six 4-hour intervals, which were assigned values in the order of “nighttime → daytime” (with the lowest code for 00:00–04:00 and the highest code for 20:00–24:00). The results indicate that a higher OT code (i.e., onset time leaning more toward daytime) corresponds to a lower predicted probability of neurological deterioration (ND), which is consistent with the circadian rhythm characteristics of stroke progression reported in previous studies [24]. This phenomenon may be attributed to circadian-driven systemic vulnerability: during nighttime and early morning, the human body experiences rhythmic disturbances in blood pressure, coagulation function, and cerebral autoregulation capacity, which are prone to causing infarct progression and secondary brain injury; meanwhile, insufficient collateral cerebral perfusion during sleep leads to a lower mean apparent diffusion coefficient value (MAV) in the infarct area (indicating more severe irreversible damage), collectively resulting in a high risk of ND. Additionally, delayed symptom detection and insufficient medical resources during nighttime onset may be another factor that further elevates the risk of ND. Second, from a modeling perspective, the LASSO regression method adopted in our model only adjusts the magnitude of variable coefficients or eliminates variables with no predictive value, and does not alter the direction of the association between variables and outcomes. The negative coefficient of OT is jointly determined by its inherent clinical association with ND and our coding strategy, rather than being an algorithm-induced bias. Therefore, we argue that OT still has statistical and clinical significance in our model construction. Regarding another variable, reductions in early MAV (< 500 × 10^− 6^ mm²/s) signify cytotoxic edema in irreversible infarct cores, rendering these regions prone to ND as edema and inflammation progress [4, 5]. Additionally, PI demonstrated high ND rates, attributable to the anatomical vulnerability of basilar perforator arteries. These small, perpendicular vessels lack collateral compensation, leading to rapid neurological decline upon occlusion [22].

In addition, different from previous studies of the same type, our research realized targeted innovations in predictive variables, particularly in the application of MAV and SBPV. Unlike the qualitative or semi-quantitative neuroimaging indicators such as infarct location and infarct volume adopted in prior research, MAV is a precise quantitative marker. In recent years, most cutting-edge literature has confirmed that the quantitative analysis of MAV in ischemic regions is more sensitive than traditional imaging indicators in determining infarct reversibility and assessing the risk of secondary brain injury, thus possessing irreplaceable clinical value in the prediction of progressive stroke. Our study further verified the predictive efficacy of MAV. Its impact on ND is indeed superior to that of similar imaging variables like infarct location and infarct volume, which is consistent with the core conclusions of previous MAV-related studies. Meanwhile, instead of using mean blood pressure, a common indicator in previous research, our study adopted SBPV as the core variable to characterize blood pressure fluctuation characteristics. According to the conclusions of relevant studies in recent years, the odds ratio (OR) of SBPV is significantly higher than that of mean blood pressure. This indicates that SBPV has a stronger ability to characterize cerebral perfusion imbalance caused by blood pressure fluctuations (hypotension leads to insufficient penumbral perfusion, while hypertension exacerbates cerebral edema and reperfusion injury), and can more accurately reflect the impact of dynamic blood pressure changes on the ND risk of patients with APCCI. The contribution of SBPV to ND risk obtained in our study is consistent with the conclusions of previous studies on blood pressure variability, and the inclusion of this indicator has also improved the performance of the model.

In the clinical utility evaluation of our model, it can be observed that within the threshold probability range of 8%-56%, the model delivers superior net benefit compared with the “treat-all” and “treat-none” strategies in clinical practice. The 8%-56% threshold range identified in this study requires hierarchical interpretation in conjunction with the disease-specific characteristics of patients with APCCI and the context of clinical intervention [25], as detailed below: (1) Clinically, the incidence of neurological deterioration (ND) in patients with APCCI ranges from 8% to 40%, and 8% serves as the demarcation point for the transition from low-risk to moderate-risk in risk stratification. Initiating basic monitoring for patients with a predicted ND risk ≥ 8% enables the early identification of potential progression risks without imposing a substantial additional medical burden. (2) Mid-range threshold (22%: peak of net benefit): The model achieves a maximum net benefit of 31% at the 22% threshold, which represents the optimal initiation node for intensified intervention. When the predicted ND risk is ≥ 22%, the implementation of comprehensive intensified management—such as completing dynamic magnetic resonance imaging (MRI) to assess infarct core progression and optimizing blood pressure management to maintain cerebral perfusion—helps balance intervention benefits and healthcare costs, making this threshold a core reference for clinical decision-making [1]. (3) Upper threshold (56%): A predicted ND risk ≥ 56% identifies the patient as part of the “extremely high-risk population”, necessitating the direct initiation of emergency-level interventions (e.g., evaluating indications for endovascular interventional therapy). Clinically, this threshold functions as a “risk warning red line”, facilitating the rapid identification of critically ill patients requiring emergent resuscitative care by clinicians. (4) Decision logic for thresholds outside the 8%-56% range: When the predicted ND risk is < 8%, intensified intervention yields no additional clinical benefits. When the predicted risk exceeds 56%, the likelihood of ND becomes nearly inevitable, and the initiation of maximum-intensity clinical intervention is thus recommended directly.

The contribution of SBPV to ND risk observed in our study is consistent with the conclusions of prior research on blood pressure variability, and the incorporation of this indicator has also improved the model’s performance. Finally, our manuscript included SRF as an original variable. This is because in the vast majority of published studies on validated predictive models, stroke-related risk factors (i.e., hypertension, diabetes, dyslipidemia, smoking, hyperhomocysteinemia, prior stroke, atrial fibrillation, valvular heart disease, and obesity) were frequently included as separate predictors, yet most of these variables failed to exhibit significant statistical associations with outcomes [8, 20, 26]. In contrast, patients with multiple comorbidities face a substantially higher risk of progressive stroke. Studies have indicated that this may be attributed to two potential mechanisms: first, the cumulative effect of multiple underlying diseases exacerbates vascular wall damage and elevates the risk of in situ thrombosis;12 s, each of these comorbidities can induce systemic inflammatory responses, and their synergistic effects trigger a neuroinflammatory cascade that causes secondary injury to the original infarcted region, thereby precipitating stroke progression [27].

The nomogram provides a visual and quantifiable tool for assessing neurological deterioration (ND) risk. For example, a 75-year-old male patient with hypertension and a smoking history. His OT was 2, SBPV was 0.1, NLR was 0.8, BNS was 3, MAV of 400, and non-pontine infarction would receive a total nomogram score of 149.5 points (age: 32, SBPV: 22, BNS: 40, NLR: 8, MAV: 37.5, OT: 10, non- pontine infarction: 0), corresponding to a 18.0% ND probability. Patients exceeding predefined risk thresholds may benefit from intensified monitoring or early endovascular therapy.

The study has several limitations. First, the sample size (testing set: 94) and ND events of our study are relatively small, which may have contributed to model overfitting and the observed decline in temporal external validation AUC. Second, the retrospective design exists selection bias and information bias (e.g., data missing), and some important research indicators are not recorded. Third, unmeasured predictors such as the High-sensitivity C-reactive protein, triglyceride-glucose index, D-dimer and homocysteine levels—known predictors of ND—were not included, potentially affecting model accuracy [9]. Fourth, as this study adopted a single-center recruitment design without external validation in non-Chinese populations or other research centers, the findings have certain limitations. Finally, subjective assessments (e.g., BNS and OT documentation), despite being performed by trained neurologists, may lack inter-rater reliability. Nevertheless, our model innovatively integrates neuroimaging biomarkers (MAV) and demonstrates reproducible performance across cohorts, providing a foundation for future multicenter prospective validation to enhance generalizability and clinical adoption.

## Conclusion

In conclusion, based on a single-center cohort with a relatively limited sample size, we developed and validated a seven-predictor nomogram for APCCI-related ND, incorporating clinical, laboratory, and imaging parameters. The model demonstrated moderate discriminative ability and calibration, with potential to guide risk-stratified management. In the future, we will employ a broader range of modeling approaches (including random forest, XGBoost, and deep learning algorithms) to develop predictive models, systematically evaluate the respective strengths and limitations of each approach, and ultimately identify the optimal model amenable to translation into real-world clinical practice. Additionally, future multicenter prospective studies should address the inherent limitations of the current study and further broaden and strengthen the generalizability of the predictive model.

## Supplementary Information

Supplementary Material 1.

## Data Availability

The datasets analyzed during this study can be obtained from the corresponding author on reasonable request.

## Abbreviations

SBPV: Systolic blood pressure variability
NIHSS: National Institutes of Health Stroke Scale
OT: Onset time
NLR: Neutrophil-to-lymphocyte ratio
MAV: Mean Apparent Diffusion Coefficient value (×10^-3^mm²/s)
PI: Pontine infarction
