# Supplementary material for: Development and validation of a prognostic model for neurological deterioration in acute posterior circulation cerebral infarction
Source: BMC Neurol. 2026 Mar 17;26:273. doi: 10.1186/s12883-026-04823-7 (PMC13107863; doi:10.1186/s12883-026-04823-7)
Supplement: Supplementary file 1 — Supplementary Material 1. [file 12883_2026_4823_MOESM1_ESM.docx]

**Development and Validation of a Prognostic Model for Neurological Deterioration in Acute Posterior Circulation Cerebral Infarction**

Zedan Guo, Qian Zhang, Jiani Wu, Li Yi

**Supplementary files for online only publication**

Table of Contents

Table of Contents........................................................................................................................2

Supplementary Methods.............................................................................................................3

Table 1 Baseline characteristics in Training and Testing cohorts (after multiple imputation)..................................................................................................................................5

Table 2 Comparison of two groups: Neurological Deteriation and Non-Neurological Deteriation (after multiple imputation).......................................................................................7

Figure S1 Heatmap of variable correlations...............................................................................9

Figure S2 LASSO regression for feature selection...................................................................10

Figure S3 Cross-validation error curve.....................................................................................11

Figure S4 Decision curve analysis (DCA) in testing set....................................................12

**Supplementary Methods**

***3D slicer operation mechanism***

3D Slicer is a free, open-source software platform widely used for medical image computing. As a clinical research tool, it supports multiple visualization methods and provides unique quantitative analysis capabilities. Researchers can configure parameters in regions of interest (ROIs) to extract detailed information from medical images. We downloaded DICOM images of diffusion-weighted imaging (DWI) sequences for each patient to local computer disks. We directly loaded DICOM images from disk into 3D Slicer. Using the Volumes module, we selected DWI and ADC maps. We set the window width to 980 and window level to 600 to enhance identification of cerebral infarction regions. Next, we manually segmented infarction regions using the Segment Editor module. 3D Slicer divides image data within ROIs into individual voxels. It automatically counts voxels and calculates ROI volume based on voxel dimensions. For DWI sequences with different b-values, the software computes ADC values per voxel using the formula: ADC = (ln(Signal_low_b)-ln(Signal_high_b))/(b_high-b_low). Signal_low_b means signal intensity at low b-value whereas signal_high_b means signal intensity at high b-value. b_high means high b-value and b_low means low b-value (typically 0-1000s/mm^2^). The software then averages voxel-based ADC values within the ROI. This mean ADC value reflects overall water molecule diffusion characteristics across the entire region.

***Data imputation and transformation***

We identified all missing data (<30% per variable) in the study. We performed multiple imputation using the mice package (v4.5.0) in R Studio. First, we filled missing values with simple replacements (mean for continuous variables, mode for categorical variables). Next, we constructed regression models for each variable with missing data, using other variables as predictors. We iteratively refined imputations by sampling new values from posterior distributions to replace temporary estimates. This process converged toward plausible values through 20 iterations. We generated one complete imputed dataset for final analysis. We log-transformed non-normally distributed variables and variables with extreme values that couldn't be removed. Finally, we standardized these transformed variables. This approach preserved distributional trends while maintaining their relationship with the outcome variable.

***LASSO regression***

We applied least absolute shrinkage and selection operator (LASSO) regression to select predictors from 14 candidate variables. We constructed the prediction model using training data. Through 10-fold cross-validation (random seed = 123), we identified the optimal penalty tuning parameter **λ**. This **λ** value minimized the mean absolute error (MAE). We selected predictors based on this optimal **λ**. Finally, we fitted the final model using the optimal **λ**. All LASSO procedures were implemented with the glmnet package in R.

**Table 1** Baseline characteristics in Training and Testing cohorts(via multiple imputation)

| **Variable** | **Training**  (n= 447) | **Testing**  (n= 94) | ***P*** |
| --- | --- | --- | --- |
| **Gender** |  |  | 0.596 |
| *Female* | 134 (30.0%) | 25 (26.6%) |  |
| *Male* | 313 (70.0%) | 69 (73.4%) |  |
| **Age** | 63.90 ± 13.83 | 62.11 ± 11.59 | 0.190 |
| **SBPV** | 0.08 ± 0.03 | 0.08 ± 0.03 | 0.612 |
| **DBPV** | 0.09 ± 0.04 | 0.09 ± 0.03 | 0.577 |
| **Baseline NHISS scores** | 2.90 ± 2.96 | 2.71 ± 3.22 | 0.610 |
| **Neurological deterioration** | 79 (17.7%) | 19 (20.2%) | 0.664 |
| **Onset time** |  |  | 0.167 |
| *0:00-04:00* | 51 (11.4%) | 9 (9.6%) |  |
| *04:00-08:00* | 68 (15.2%) | 22 (23.4%) |  |
| *08:00-12:00* | 85 (19.0%) | 23 (24.5%) |  |
| *12:00-16:00* | 64 (14.3%) | 14 (14.9%) |  |
| *16:00-20:00* | 96 (21.5%) | 15 (16.0%) |  |
| *20:00-24:00* | 83 (18.6%) | 11 (11.7%) |  |
| **NLR** | 3.99 ± 4.04 | 4.70 ± 4.59 | 0.168 |
| **LDL-C** | 3.19 ± 0.89 | 3.39 ± 1.10 | 0.092 |

**Table 1** Continued

| **Variable** | | **Training**  (n= 450) | | **Testing**  (n= 91) | ***P*** |
| --- | --- | --- | --- | --- | --- |
| **Infarct volume** | 1,955.38 ± 6,213.95 | | 5,755.34 ± 11,847.13 | | 0.003 |
| **Mean ADC value** | | 478.30 ± 77.11 | | 485.17 ± 81.41 | 0.454 |
| **Pontine infarction** | |  | |  | >0.999 |
| *No* | | 265 (59.3%) | | 56 (59.6%) |  |
| *Yes* | | 182 (40.7%) | | 38 (40.4%) |  |
| **Infarction subtype** | |  | |  | 0.211 |
| *Large-artery atherosclerosis* | | 142 (31.8%) | | 38 (40.4%) |  |
| *Cardioembolism* | | 36 (8.1%) | | 2 (2.1%) |  |
| *Small-vessel occlusion* | | 238 (53.2%) | | 47 (50.0%) |  |
| *Other determined etiology* | | 8 (1.8%) | | 2 (2.1%) |  |
| *Undermined etiology* | | 23 (5.1%) | | 5 (5.3%) |  |
| **Stroke risk factors** | |  | |  | 0.637 |
| *0 (None)* | | 43 (9.6%) | | 8 (8.5%) |  |
| *1 (Mild)* | | 235 (52.6%) | | 45 (47.9%) |  |
| *2 (Moderate)* | | 150 (33.6%) | | 38 (40.4%) |  |
| *3 (Severe)* | | 19 (4.3%) | | 3 (3.2%) |  |
| **TG** | | 1.63 ± 1.12 | | 1.60 ± 0.82 | 0.801 |

SBPV，systolic blood pressure variability; DBPV, diastolic blood pressure variability; NIHSS, National Institutes of Health Stroke Scale; NLR, neutrophil-to-lymphocyte ratio; LDL-C, low density lipoprotein cholesterol; TG; triglyceride.

**Table 2** Comparison of two groups: Neurological Deteriation and Non-Neurological Deteriation (via multiple imputation)

| **Variable** | **Neurological Deterioration**  (n= 98) | **Non-Neurological Deterioration**  (n= 443) | **P** |
| --- | --- | --- | --- |
| **Gender** |  |  | 0.941 |
| *Female* | 28 (28.6%) | 131 (29.6%) |  |
| *Male* | 70 (71.4%) | 312 (70.4%) |  |
| **Age** | 66.28 ± 12.03 | 63.00 ± 13.71 | 0.019 |
| **SBPV** | 0.09 ± 0.03 | 0.08 ± 0.03 | 0.113 |
| **DBPV** | 0.09 ± 0.04 | 0.09 ± 0.04 | 0.525 |
| **Baseline NIHSS scores** | 4.17 ± 2.97 | 2.58 ± 2.94 | <0.001 |
| **Onset time** |  |  | 0.412 |
| *0:00-04:00* | 11 (11.2%) | 49 (11.1%) |  |
| *04:00-08:00* | 20 (20.4%) | 70 (15.8%) |  |
| *08:00-12:00* | 22 (22.4%) | 86 (19.4%) |  |
| *12:00-16:00* | 17 (17.3%) | 61 (13.8%) |  |
| *16:00-20:00* | 14 (14.3%) | 97 (21.9%) |  |
| *20:00-24:00* | 14 (14.3%) | 80 (18.1%) |  |
| **NLR** | 5.14 ± 5.83 | 3.89 ± 3.64 | 0.043 |
| **LDL-C** | 3.25 ± 0.95 | 3.22 ± 0.93 | 0.801 |
| **Infarct volume** | 2,376.14 ± 9,406.22 | 2,668.62 ± 7,183.26 | 0.773 |
| **Mean ADC value** | 447.35 ± 78.15 | 486.61 ± 76.04 | <0.001 |
| **Pontine infarction** |  |  | <0.001 |
| *No* | 41 (41.8%) | 280 (63.2%) |  |
| *Yes* | 57 (58.2%) | 163 (36.8%) |  |
| **Infarction subtype** |  |  | 0.030 |

**Table 2** Continued

| **Variable** | | **Neurological Deterioration**  (n= 98) | **Non-Neurological Deterioration**  (n= 443) | | | **P** |
| --- | --- | --- | --- | --- | --- | --- |
| *Large-artery atherosclerosis* | | 36 (36.7%) | 144 (32.5%) | | |  |
| *Cardioembolism* | 3 (3.1%) | | | 35 (7.9%) |  | |
| *Small-vessel occlusion* | 58 (59.2%) | | | 227 (51.2%) |  | |
| *Other determined etiology* | 1 (1.0%) | | | 9 (2.0%) |  | |
| *Undermined etiology* | 0 (0.0%) | | | 28 (6.3%) |  | |
| **Stroke risk factors** |  | | |  | 0.324 | |
| *0 (None)* | 5 (5.1%) | | | 46 (10.4%) |  | |
| *1 (Mild)* | 57 (58.2%) | | | 223 (50.3%) |  | |
| *2 (Moderate)* | 32 (32.7%) | | | 156 (35.2%) |  | |
| *3 (Severe)* | 4 (4.1%) | | | 18 (4.1%) |  | |
| **TG** | 1.63 ± 1.15 | | | 1.62 ± 1.06 | 0.957 | |

SBPV，systolic blood pressure variability; DBPV, diastolic blood pressure variability; NIHSS, National Institutes of Health Stroke Scale; NLR, neutrophil-to-lymphocyte ratio; LDL-C, low density lipoprotein cholesterol; TG; triglyceride.

**Figs. 1** Heatmap of variable correlations


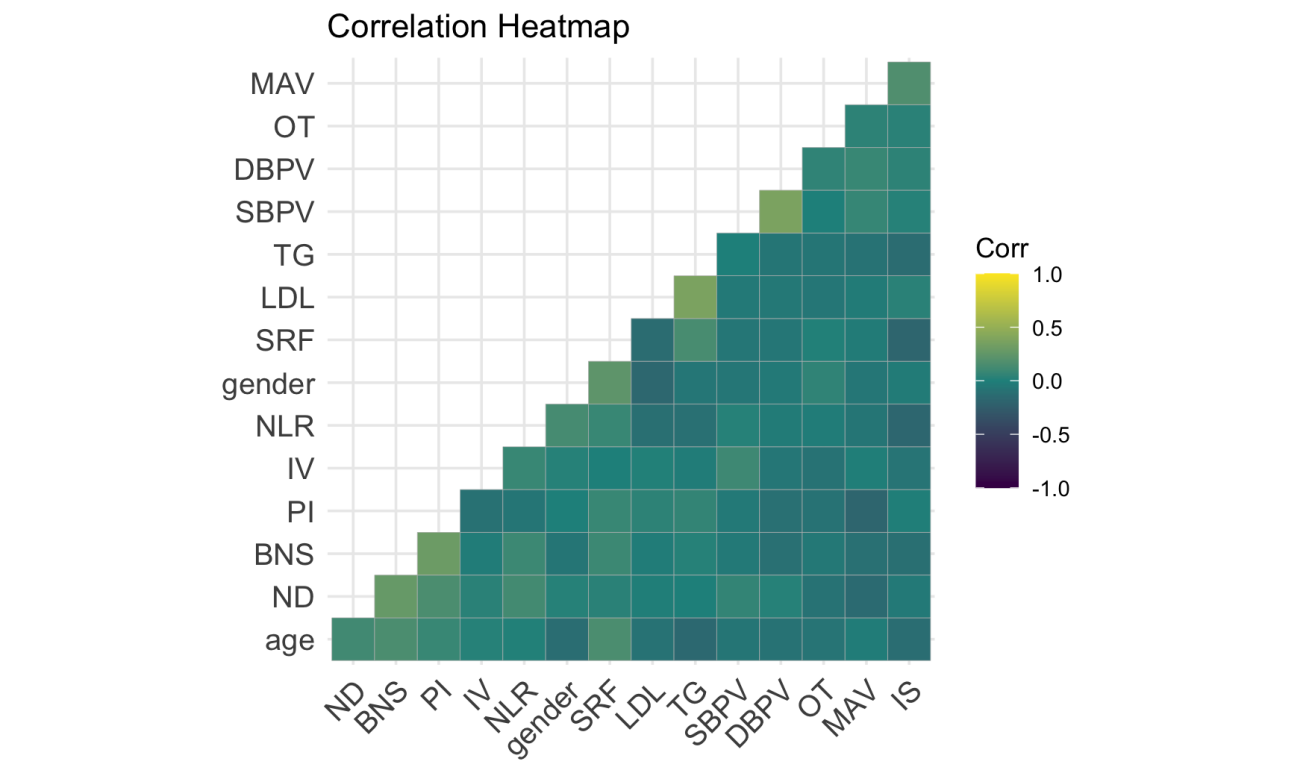


**Legend text:** Correlation analysis results of the 14 predictive variables. Continuous variables used Pearson correlation. Ordinal or non-normally distributed variables used Spearman correlation.(Yellow: positive correlation; Blue: negative correlation). SBPV and DBPV showed a moderate correlation ( |r| = 0.37). No significant correlations were observed among other variables.

**Figs. 2** LASSO regression for feature selection


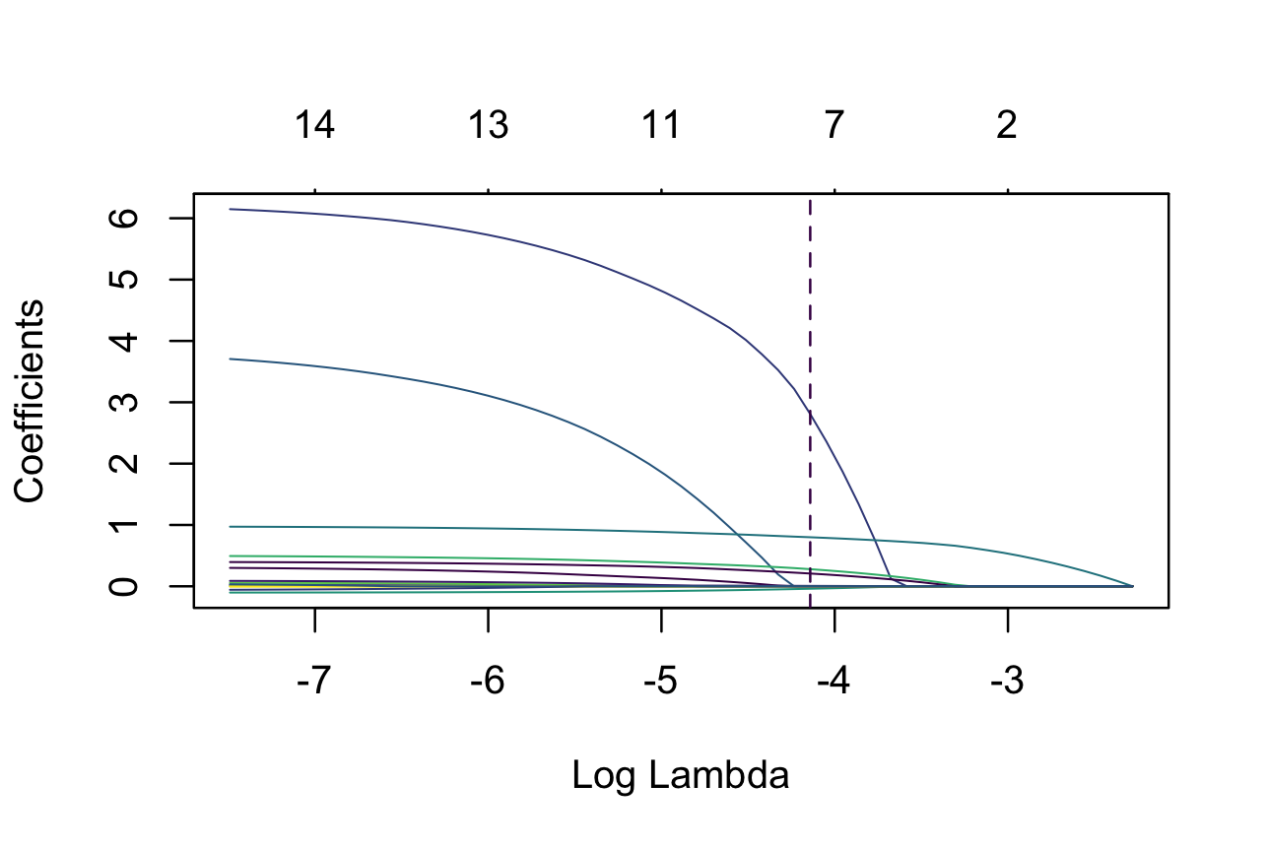


**Legend text:** The optimal **λ** value was identified through 10-fold cross-validation (log-transformed **λ** = 0.015). Seven variables with non-zero coefficients were selected: age, SBPV, baseline NHISS score, onset time, NLR, mean ADC value, pontine infarction. Seven variables (gender, DBPV, LDL, TG, infarct volume, stroke risk factors and infarction subtype) were excluded. The model intercept was -2.374389404.

**Figs. 3** Cross-validation error curve


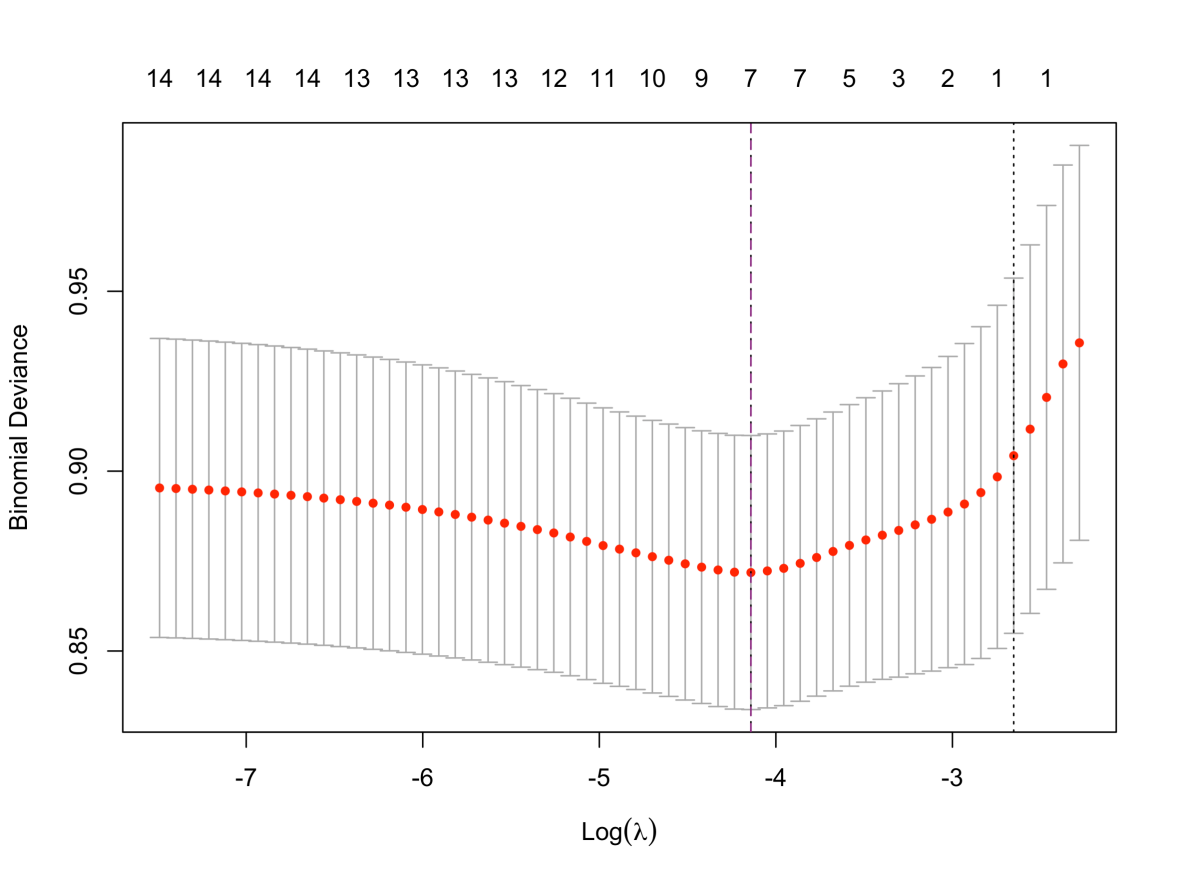


**Legend text:** Vertical dashed lines indicate the **λ** value corresponding to the minimum mean squared error (**λ** = 0.015). At this **λ** value, the model achieved optimal predictive performance while maintaining variable simplification.

**Figs. 4** Decision curve analysis (DCA) in testing set


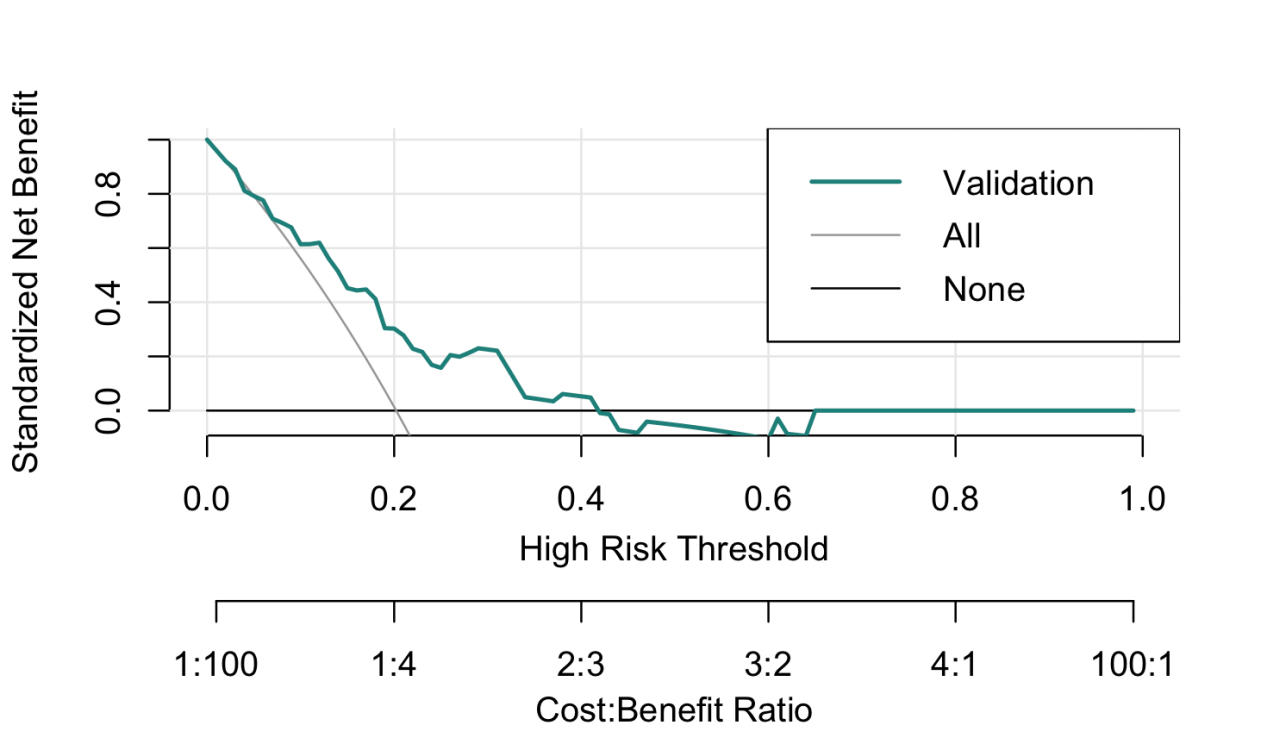


**Legend text:** Within the threshold probability range of 10–41%, the predictive model demonstrated higher clinical net benefit compared to the "Treat All" or "Treat None" strategies.
